# Erratum to: Genome wide association mapping of *Pyrenophora teres* f. *maculata* and *Pyrenophora teres* f. teres resistance loci utilizing natural Turkish wild and landrace barley populations

**DOI:** 10.1093/g3journal/jkab280

**Published:** 2021-09-22

**Authors:** Shaun J Clare, Arzu Çelik Oğuz, Karl Effertz, Roshan Sharma Poudel, Deven See, Aziz Karakaya, Robert S Brueggeman

*G3* 2021, 1–13

An incorrect manuscript was inadvertently published under DOI 10.1093/g3journal/jkab269 due to production error. The definitive uncorrected manuscript version of the above stated article has now been reinstated.

